# Antimycobacterial drug discovery using Mycobacteria-infected amoebae identifies anti-infectives and new molecular targets

**DOI:** 10.1038/s41598-018-22228-6

**Published:** 2018-03-02

**Authors:** Valentin Trofimov, Sébastien Kicka, Sabrina Mucaria, Nabil Hanna, Fernando Ramon-Olayo, Laura Vela-Gonzalez Del Peral, Joël Lelièvre, Lluís Ballell, Leonardo Scapozza, Gurdyal S. Besra, Jonathan A. G. Cox, Thierry Soldati

**Affiliations:** 1 Department of Biochemistry, Faculty of Science, University of Geneva, Geneva, Switzerland; 2 Pharmaceutical Biochemistry/Chemistry, School of Pharmaceutical Sciences, University of Geneva, Geneva, Switzerland; 3 GSK, Severo Ochoa 2, 28760 Tres Cantos, Madrid, Spain; 4 School of Biosciences, University of Birmingham, Edgbaston, Birmingham, UK; 5 School of Life & Health Sciences, Aston University, Birmingham, UK; 6 Present Address: Institut Pasteur de Lille, Lille, France

## Abstract

Tuberculosis remains a serious threat to human health world-wide, and improved efficiency of medical treatment requires a better understanding of the pathogenesis and the discovery of new drugs. In the present study, we performed a whole-cell based screen in order to complete the characterization of 168 compounds from the GlaxoSmithKline TB-set. We have established and utilized novel previously unexplored host-model systems to characterize the GSK compounds, i.e. the amoeboid organisms *D. discoideum* and *A. castellanii*, as well as a microglial phagocytic cell line, BV2. We infected these host cells with *Mycobacterium marinum* to monitor and characterize the anti-infective activity of the compounds with quantitative fluorescence measurements and high-content microscopy. In summary, 88.1% of the compounds were confirmed as antibiotics against *M*. *marinum*, 11.3% and 4.8% displayed strong anti-infective activity in, respectively, the mammalian and protozoan infection models. Additionally, in the two systems, 13–14% of the compounds displayed pro-infective activity. Our studies underline the relevance of using evolutionarily distant pathogen and host models in order to reveal conserved mechanisms of virulence and defence, respectively, which are potential “universal” targets for intervention. Subsequent mechanism of action studies based on generation of over-expresser *M. bovis* BCG strains, generation of spontaneous resistant mutants and whole genome sequencing revealed four new molecular targets, including FbpA, MurC, MmpL3 and GlpK.

## Introduction

Tuberculosis (TB) remains one of the most serious health-threats worldwide with 8 million cases of new infection annually. In the last few decades, various steps were taken to reduce the burden of TB. However, despite the decline in prevalence and mortality, the death count still remains astonishingly high. In 2014, TB killed 1.5 million people, which makes it one of the most deadly bacterial infectious diseases worldwide^1^. Furthermore, the emergence of multidrug-resistant *Mycobacterium tuberculosis* (*Mtb*) has gradually rendered current frontline TB therapy inefficient. To reduce the TB burden, it is absolutely necessary to discover new anti-TB drugs and to improve methodologies of TB detection and treatment. There are several drug candidates at different stages of the development pipeline, including TB-354 in Phase I clinical trial (WHO 2015).

The discovery of new drugs faces many challenges. For example, the low metabolic rate of *Mtb* significantly decreases bactericidal activity of antibiotics^2^. Drug efficacy is also reduced by the highly impermeable hydrophobic mycobacteria cell wall, intracellular degradation and the activity of efflux pumps of the host as well as the bacterium^3^. Additionally, *Mtb* is able to manipulate the environment inside the alveolar macrophages via modulation of membrane trafficking^4,5^, of autophagy^6,7^, by alteration of the signalling pathways^8^ and by induction of programmed cell death^9,10^. Finally, *Mtb* causes the formation of granulomas that both help contain and facilitate dissemination of the infection^11,12^. Many drugs with a clinically relevant high bactericidal potency show comparatively insignificant effects in *in vivo* infection model systems^13^.

The high attrition rate may also indicate involvement of additional parameters, such as the influence of host-drug and host-pathogen interactions^14^. The host partially shields the bacteria from anti-TB treatment, decreasing the efficiency of various antibiotics^15,16^. Lastly, one cannot ignore the changes of mycobacterial metabolism during infection^17^, specifically initiation of a dormant, persistent stage^18^ and *Mtb* switching from glycolysis to fatty acids metabolism^19–21^.

The influence of the host might also be beneficial to help eradicate the infection by host and pathogen-mediated activation of prodrugs and other anti-infective processes, such as ‘nutritional immunity’^22^. Compounds with such activity belong to the ‘defence boosters’ category and act via the activation of the protective mechanisms of the host cell. This approach may provide a means by which to discover compounds that act on a broad range of infectious diseases.

Overall, this low success of *in vitro* and target-based screening strategies recently led to a resurgence of whole-cell phenotypic screening^23^. Unlike target-based approaches, this strategy takes into account many of the aforementioned *in vivo* parameters from the beginning of the screening process. The drawbacks, however, include the lack of predefined mechanisms of action of the drug-candidates.

Whole-cell phenotypic screening can be performed either directly with *Mtb* and human CD14 positive peripheral blood mononuclear cells or by using pathogen and host substitutes. Selecting a model is a fine balance between experimental costs, ethical, genetic and experimental tractability. *M. marinum* has recently been adopted in many studies^24^. Mammalian hosts offer high phenotypic relevance to human TB, and are therefore better suited to later stages of drug discovery and development. On the other hand, simpler organisms provide less ethical constrains and statistical variations, ease of manipulation and high throughput application in early drug discovery as well as in focused mechanism of action studies and target verification. In recent years, a variety of host model systems have been used for the study of TB, including primates^25^, rodents^26,27^, zebrafish^28^, Drosophila^29^ and protozoans^30^. Two major amoeba systems, *Acanthamoeba castellanii* and *Dictyostelium discoideum*, have been used to study the course of infection with various pathogenic bacteria^31^, including screens with *M. marinum* and other virulent mycobacteria^32^. They are also well-established models of microbial infections and host-pathogen interactions^31,33–36^, and drug discovery^37–39^. In addition, the compact and haploid genome of *D. discoideum* provides expanded opportunities for fast and effective genetic engineering.

In the TB drug discovery pipeline, phenotypic screening is followed by molecular target identification through mechanism of action studies. There are two main non-exclusive strategies to discovering the molecular targets of compounds: direct biochemical methods and genetic interactions methods. The direct approach often involves immobilization of the interacting protein, a “fishing” procedure, and crosslinking of the protein to the target molecule and purification of the covalent complex. Genetic interactions methods involve analysis of changes of genetic expression patterns in the presence or absence of compound. In bacteria, molecular targets are routinely identified by generation of spontaneously resistant mutants, verified by whole-genome sequencing or by the use of over-expresser mutants. Generally, the integration of multiple complementary methodologies is required to fully solve the problem of target identification.

In the present study, we decided to address the lack of data related to the *in vivo* characterization of compounds from the GSK TB-set, and performed a whole cell-based phenotypic screen, as well as more traditional growth and cytotoxicity assays. The screening was followed by molecular target identification studies.

## Results

Firstly, compounds were characterized in fluorescence-based phenotypic assays with the use of *M. marinum* and protozoan/mammalian cell systems. The compounds from the GSK TB library were scrutinized for cytotoxic, antibiotic and anti-infective properties.

### Over 70% of the compounds from the GSK TB-set show antibiotic activities against *M. marinum*

**Figure 1:**
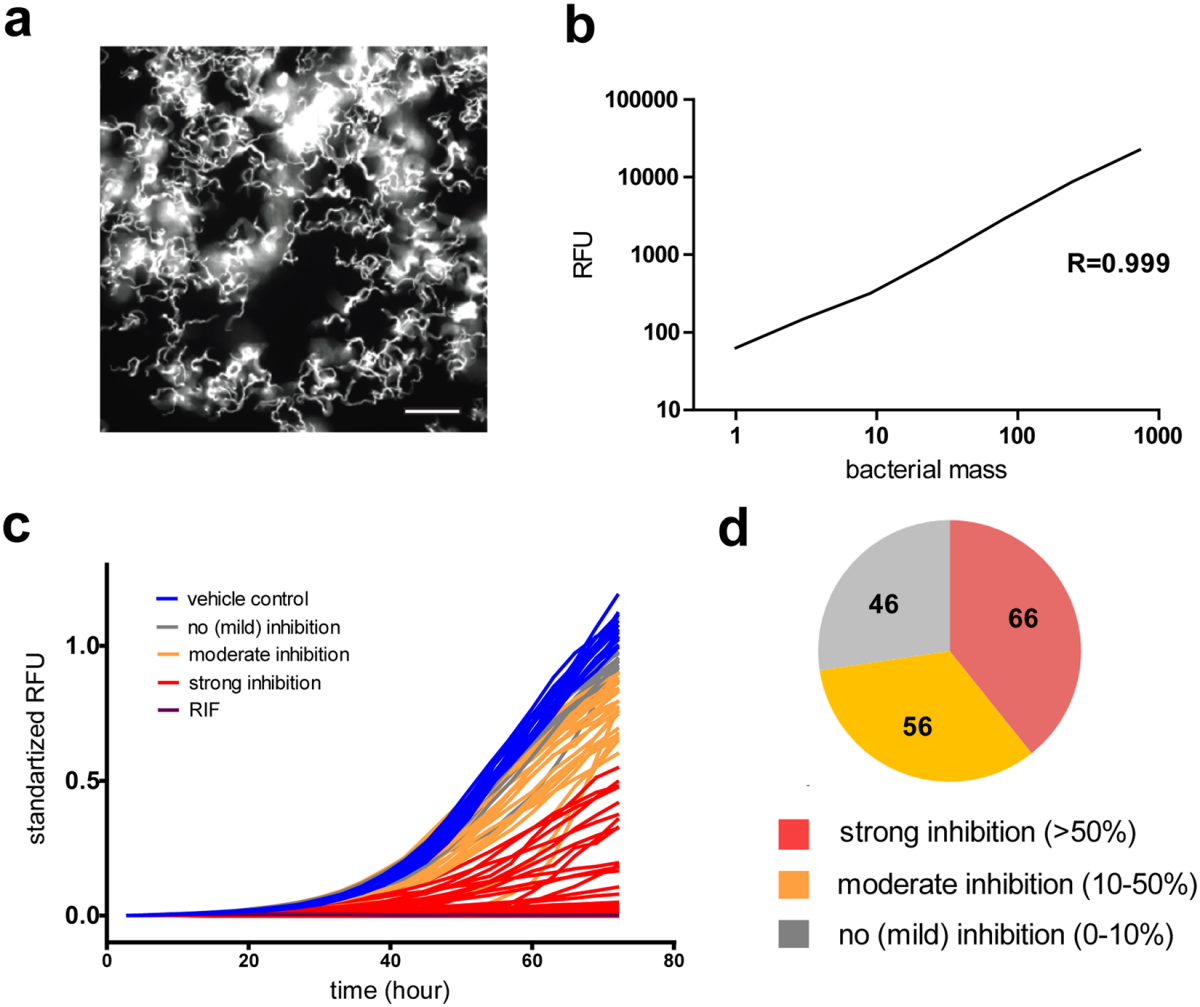
Characterization of antibiotic activities of GSK TB-set compounds. (**a**) Fluorescent confocal microscopy of 10^5^ *M. marinum* msp12::GFP, 72 hours incubation time in 7H9 broth. The scale bar corresponds to 20 µm. (**b**) Correlation between fluorescence intensity and serial dilution. Serial dilutions of *M. marinum* msp12::GFP, were transferred to 96-well plate. Dilution factor is 3. A relative density unit corresponds to 10^5^ bacteria. (**c**) Growth kinetics of *M. marinum* msp12::GFP in 7H9 broth and in the presence compounds from GSK TB-set (color-coded green) of vehicle control (blue), supplemented with 10 μM amikacin (purple), 10 μM rifabutin (red) representative experiment from a series with similar outcome. 10^5^ *M. marinum* expressing GFP were transferred to the wells. Fluorescence intensities were measured for 72 hours every 3 hours. (**d**) Pie chart representing summarized results of growth inhibitory activity of 168 compounds in *M. marinum* growth assay. The analyzed set included compounds that displayed inhibition of more than 90% RFU (red), inhibition between 30 and 90% (orange), compounds that showed less than 30% inhibition (grey).

We established an assay to measure the antibiotic and bacteriostatic activity of compounds against *M. marinum* in the absence of the host (Figs 1A–C, S1). Compounds at 10 μM in DMSO, a concentration similar to the one used in the original BCG-based screening^40^, were transferred to 96-well plates containing 10^5^ GFP-expressing *M. marinum* per well. Growth was monitored for 35–72 hours, and the total fluorescence intensity was used as a proxy to quantify the bacterial mass. Compared to DMSO alone, we observed reproducible 10–100% decrease of the fluorescence intensity for 122 compounds out of 168, with 66 showing more than 50% inhibition, while 56 displayed 10–50% inhibition. Interestingly, in this antibiotic assay, 46 out of 168 compounds were found to have negligible effect (less than 10% inhibition) on bacterial growth (Tables 1 and S1, Fig. 1D). We also verified the compounds’ antibiotic activity using the luxCDABE-expressing auto-bioluminescent *M. marinum* strain. Compared to the vehicle control, we observed 81–100% decrease of the fluorescence intensity for 111 compounds out of 168, while 37 displayed 11–80% inhibition. Among 168 compounds, 20 were found to have negligible effect (less than 10% inhibition) on bacterial growth (Tables 1 and S1, and see Discussion for a more detailed comparison of the two sets of results). These two assays allowed us to characterize antibacterial activities independent from host-pathogen interactions. Data derived from this assay served as a reference point, since broth-based phenotypic screening is one of the core techniques in drug discovery and was used for initial establishment of the GSK TB-set^40^. The fact that the majority of the compounds decreased the rate of *M. marinum* growth indicates high potential for using this mycobacterial strain as a substitute for *Mtb* in classical antibiotic assays. Lack of significant bactericidal activity for certain compounds might be explained by genetic and metabolic differences between *M. marinum* and *Mtb*.

**Table 1:** Selected hits from Table S1 detected by screening the GSK TB-set.

GSK ID	plate coordinate	Antibiotic assays			Growth inhibition/Cytotoxicity assays			Intracellular infection assays					Resistant mutants	hit in BV2/M. marinum assay	Hits A. castellanii/M. marinum assay
		*M*. *marinum* luxCDABE	*M. marinum GFP*		*D. discoideum GFP-ABD*		BV2 cells	*A*. *castellanii*/*M*. *marinum*		BV2/*M*. *marinum*			*M. bovis* BCG		
		normalised growth, control = 1.00					True/False	normalised growth, control = 1.00					Potential target	True/False	True/False
		10 µM, mean	10 µM, mean	stdev	10 µM, mean	stdev	10 µM mean	10 µM, mean	stdev	10 µM, mean	stdev	IC50			
GSK1055950A	2B04	0,00	−0,01	0,01	1,07	0,10	FALSE	0,24	0,12	0,33	0,24	2,84	ND	TRUE	TRUE
GSK1829732A	2F05	0,00	0,06	0,04	1,00	0,12	FALSE	0,56	0,08	0,34	0,17	3,52	ND	TRUE	FALSE
GSK1829676A	2C05	0,00	0,03	0,04	0,75	0,12	FALSE	0,56	0,25	0,35	0,20	−1,36	ND	TRUE	FALSE
GSK2111534A	2G07	0,00	0,06	0,05	0,83	0,14	FALSE	0,51	0,12	0,36	0,11		DHFR	TRUE	FALSE
GSK1829819A	2H05	0,00	0,08	0,06	1,08	0,10	FALSE	0,66	0,12	0,37	0,16	3,90	ND	TRUE	FALSE
GSK353496A	2B10	0,00	0,04	0,05	0,87	0,15	FALSE	0,57	0,09	0,39	0,15	7,84	DHFR	TRUE	FALSE
GSK3011724A	2C02	0,14	0,08	0,03	0,99	0,12	FALSE	−0,32	0,22	0,40	0,05	4,44	Target identified	TRUE	TRUE
GSK1829736A	2C06	0,00	0,04	0,05	0,93	0,09	FALSE	0,60	0,19	0,41	0,30	2,21	ND	TRUE	FALSE
GSK353069A	1B06	0,00	0,01	0,02	0,99	0,10	FALSE	0,47	0,05	0,42	0,12	1,31	No Mutants	TRUE	TRUE
GSK1829728A	2G05	0,00	0,10	0,02	1,01	0,14	FALSE	0,78	0,39	0,43	0,08	4,03	ND	TRUE	FALSE
GSK1925843A	2H04	0,00	0,70	0,11	1,10	0,15	FALSE	1,12	0,39	0,43	0,29	3,83	ND	TRUE	FALSE
GSK1985270A	3G02	0,00	0,09	0,03	0,99	0,11	FALSE	−0,06	0,06	0,45	0,16	4,88	ND	TRUE	TRUE
GSK498315A	1.00E+05	0,00	0,06	0,05	0,81	0,15	FALSE	0,56	0,02	0,47	0,10	5,95	ND	TRUE	FALSE
GSK1829729A	2D05	0,00	0,04	0,03	0,86	0,13	FALSE	0,52	0,08	0,48	0,40	0,08	ND	TRUE	FALSE
GSK695914A	1B07	0,00	0,05	0,08	0,98	0,08	FALSE	0,63	0,06	0,48	0,16	7,82	ND	TRUE	FALSE
GW861072X	2C09	0,00	0,05	0,06	0,68	0,08	FALSE	0,54	0,14	0,48	0,24	7,99	ND	TRUE	FALSE
GSK1829671A	2A05	0,00	0,06	0,10	1,21	0,22	FALSE	0,66	0,14	0,49	0,31	7,47	ND	TRUE	FALSE
GSK254610A	1H02	0,00	0,05	0,06	0,88	0,60	FALSE	0,87	0,13	0,50	0,03		ND	TRUE	FALSE
GR223839X	2F10	0,00	0,08	0,10	0,64	0,06	FALSE	0,70	0,31	0,50	0,30	7,35	ND	TRUE	FALSE
GSK1829820A	2A06	0,00	0,05	0,09	1,10	0,15	FALSE	0,49	0,14	0,65	0,63	−1,58	QcrB	FALSE	TRUE
GW369335X	3C02	0,20	0,14	0,06	0,97	0,02	FALSE	0,37	0,23	0,71	0,27	4,87	DHFR	FALSE	TRUE
GW664700A	2.00E+11	0,07	0,33	0,21	−0,01	0,03	FALSE	−0,11	0,09	0,91	0,26	4,16	ND	FALSE	TRUE
GSK831784A	1H07	0,00	0,10	0,12	0,98	0,50	FALSE	0,20	0,24	0,97	0,34	14,96	MurC	FALSE	TRUE

### Confirmation of the low cytotoxicity of compounds from the GSK TB-set on murine microglial cells

**Figure 2:**
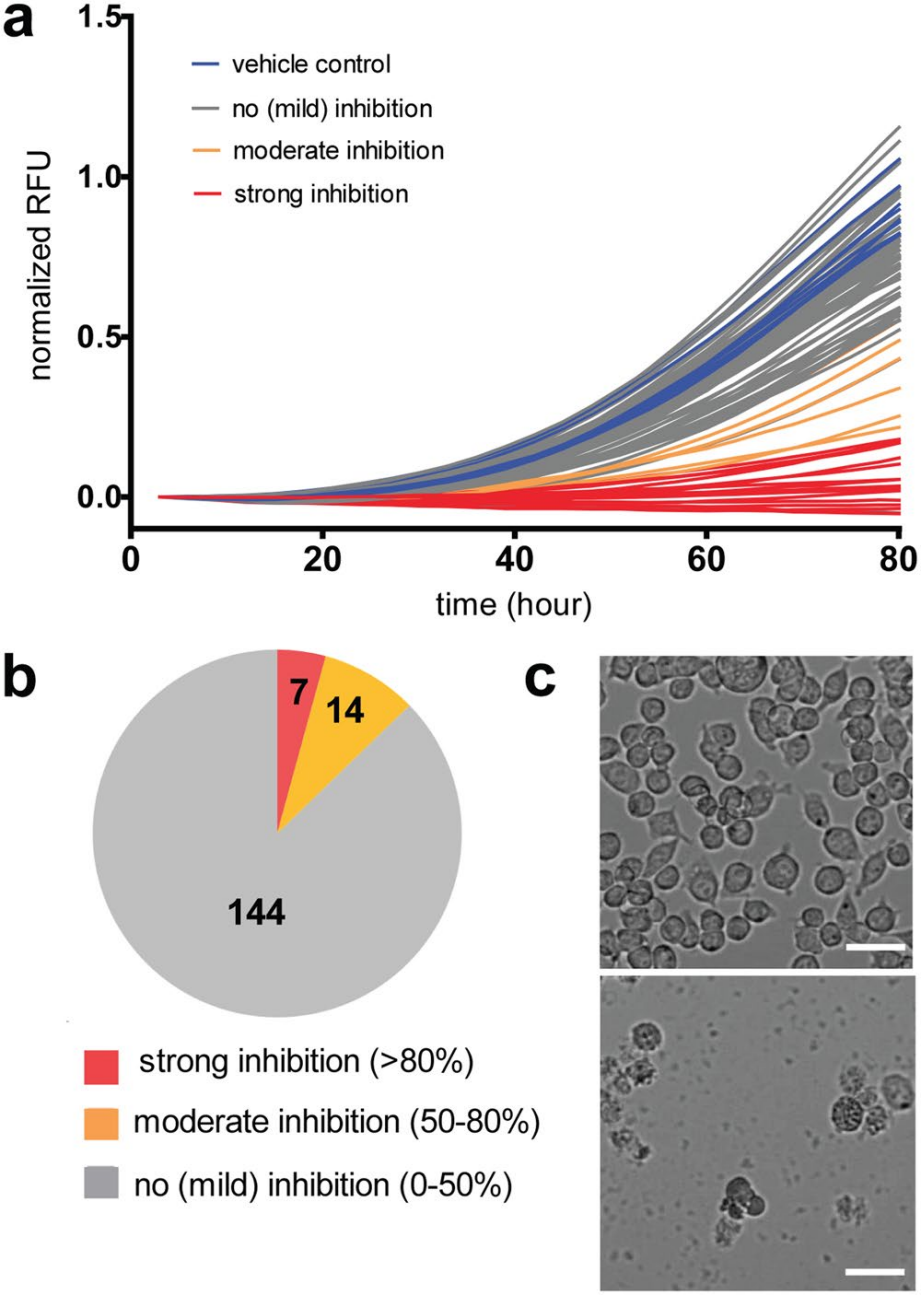
Characterization of cytotoxic activities of GSK TB-set compounds. (**a**) Growth kinetics of *D. discoideum GFP-ABD* in HL5c medium and in the presence of 10 μM corresponded compounds from GSK TB set. (**b**) Pie chart representing summarized results of growth inhibitory activity of 168 compounds in BV2 growth assay. The analysed set included compounds that displayed inhibition of more than 80% normalized RFU (red), moderate inhibition between 50 and 80% (orange), compounds that showed less than 50% inhibition (grey). (**c**) Bright-field microscopy of BV2 cells in the presence of non-cytotoxic compounds (upper image) of cytotoxic compounds (lower image. 10^5^ BV2 cells were transferred to the wells, the measurements were taken after 60 hours of incubation. The scale bar corresponds to 500 µm.

In order to estimate the inhibitory activity of each compound on host cell growth, we decided to use both amoeba (Fig. 2A) and mammalian cell-based assays. For the latter, high-content phase contrast microscopy of murine microglial BV2 cells was used (Figs 2B,C, S2). In brief, 10 μM of each compound in DMSO was transferred to 96-well plates with each well containing 10^5^ BV2 cells, and incubated for 60 to 72 hours at 37 °C. Distinguishing between growth inhibition and cytotoxicity was performed by visual inspection as an end-point measurement after 60 hours of incubation in the presence of the compound. Results indicated that at 10 μM, 158 compounds did not show significant inhibition of growth (<50% inhibition) on murine microglial cells, we also detected 5 clearly cytotoxic hits, and 5 fell into the category of mildly cytotoxic compounds.

### Confirmation of the low cytotoxicity of compounds from the GSK TB-set on the protozoan organism *D. discoideum*

Basic cellular functions regulated by housekeeping genes are conserved between *D. discoideum* and mammalian cells. Therefore, to assess its suitability to predict the cytotoxicity of compounds, we established a *D. discoideum*-based assay to measure toxic and growth inhibitory activities of compounds (Figs 2A, S3A,B). *D. discoideum* cells expressing GFP-ABD were used for the assay, the total fluorescence signal was used for cell quantification, as it is proportional to cell count. The linear relationship between cell biomass and the fluorescence/luminescence signals was confirmed by the determination of coefficients of correlation between CFU counting, fluorescence measurements (RFU), bioluminescence (RLU) and, and optical density (OD) in *M. marinum* cultures (Fig. S4). Distinguishing between cytotoxic and growth inhibitory properties of compounds was performed by visual inspection. Cells were searched for phenotypic characteristics of cell death such as membrane permeability and loss of substrate attachment. Among the analysed compounds at a 10 µM concentration, 144 compounds out of 168 (85.7%) did not display cytotoxic properties (threshold of inhibition <50%), we also detected 4 (2.3%) clearly cytotoxic hits (inhibition >80%), and 20 fell into the category of mildly cytotoxic compounds (Fig. 2B). Presence of cytotoxic activity for 2.3% of compounds might be explained by genetic and metabolic differences between *D. discoideum* and the mammalian HepG2 cell line used in the original cytotoxicity test^40^. We found 3 hits with cytotoxic activities both in BV2 and *D. discoideum* growth assays.

### Less than 5% of the *compounds* from the GSK TB-set exert strong anti-infective activity against *M. marinum* inside the *A. castellanii* protozoan host

**Figure 3:**
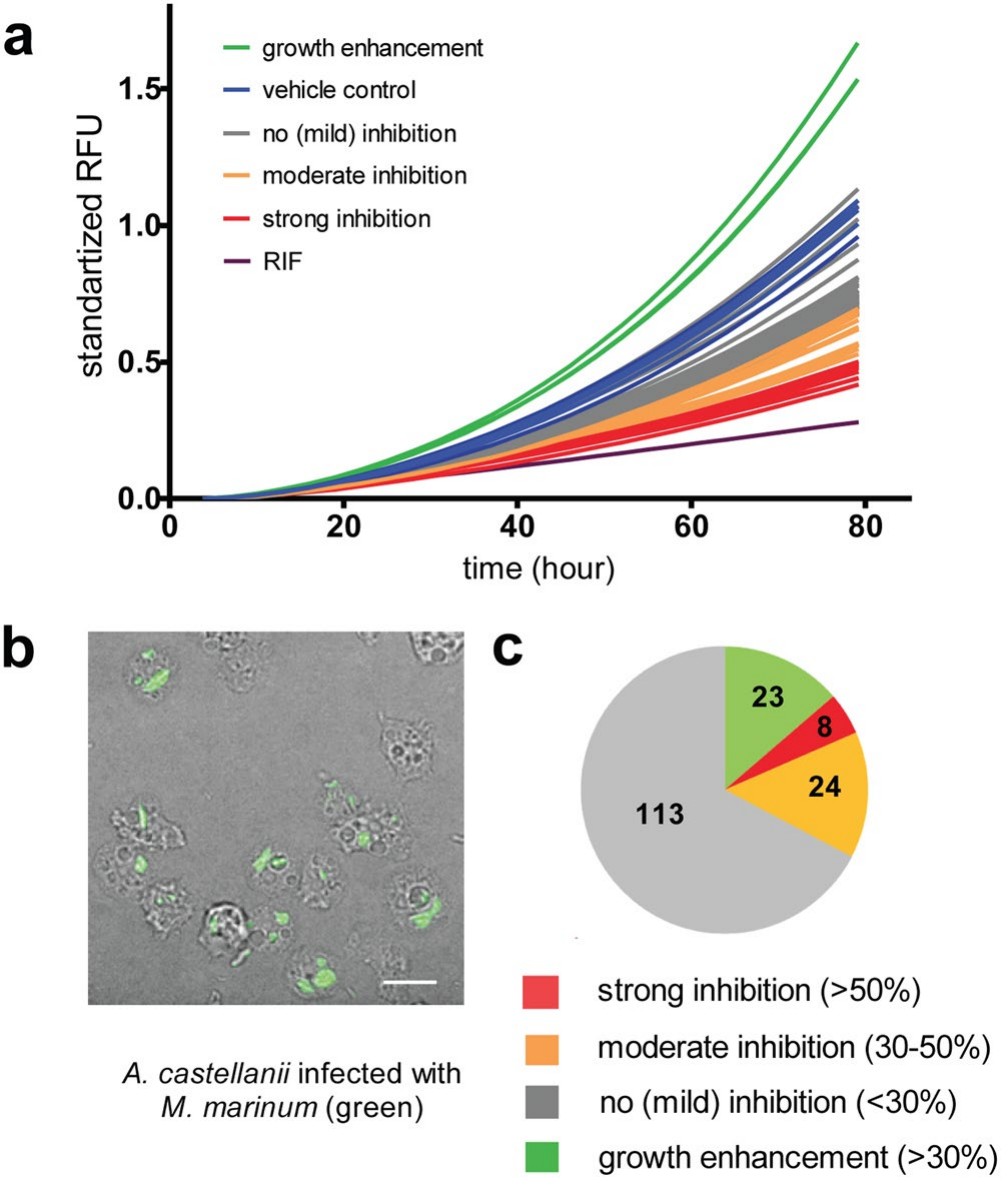
Characterization of anti-infective activities of GSK TB-set compounds in protozoan infection assay. (**a**) Growth kinetics of *M. marinum* msp12::GFP in *A. castellanii* infection, 10^5^ *A. castellanii* were transferred to the wells, 60 hours incubation time, PYG medium and in the presence of 10 μM corresponded compounds from GSK TB set. (**b**). Phase contrast/fluorescence microscopy of *A. castellanii* infection with *M. marinum* msp12::GFP. The scale bar corresponds to 30 μm. (**c**) Pie chart representing summarized results of growth inhibitory activity of 168 compounds in *A. castellanii* infection assay. The analyzed set included compounds that displayed inhibition of more than 50% normalized RFU (red), moderate inhibition between 30 and 50% (red), compounds that showed less than 30% inhibition (grey) and compounds that displayed increase of RFU by more than 30% (green). The scale bar corresponds to 200 µm.

We characterized the *in vivo* activity of the 168 compounds from the GSK TB-set during infection of *A. castellanii* with *M. marinum* (Figs 3A,B, S5). The assay is aimed at estimating the intracellular activity of the compounds, a crucial characteristic in drug discovery and development. *A. castellanii* cells were infected with GFP-expressing *M. marinum*. Intracellular bacterial growth was monitored by measuring the total fluorescence of GFP using a Synergy MX fluorescence plate reader^37^. Extracellular growth of *M. marinum* was inhibited by the presence of 10 μM amikacin in the medium. This compound is known to have low cellular permeability^16^ and, at this concentration, exerts selective bacteriostatic activity on bacteria outside host cells. Results of experiments performed as a biological triplicate are presented in Table S1 and classical kinetic curves of intracellular bacterial growth from the three plates are presented in Figs 3B and S4. The majority of the compounds decreased the rate of intracellular mycobacterial growth. However, only eight compounds (4.8%) possessed strong anti-infective activity, with more than 50% growth inhibition (Fig. S6B). Twenty-four showed moderate inhibition between 30 and 50% (Fig. 3C). Interestingly, twenty-three compounds (13.7%) also demonstrated clear pro-infective activity, resulting in more than 30% increase of bacterial growth over the infection in the presence of the vehicle control.

### Comparative anti-infective effects of compounds from the GSK TB-set on *M. marinum* growth in BV2 microglial cells

**Figure 4:**
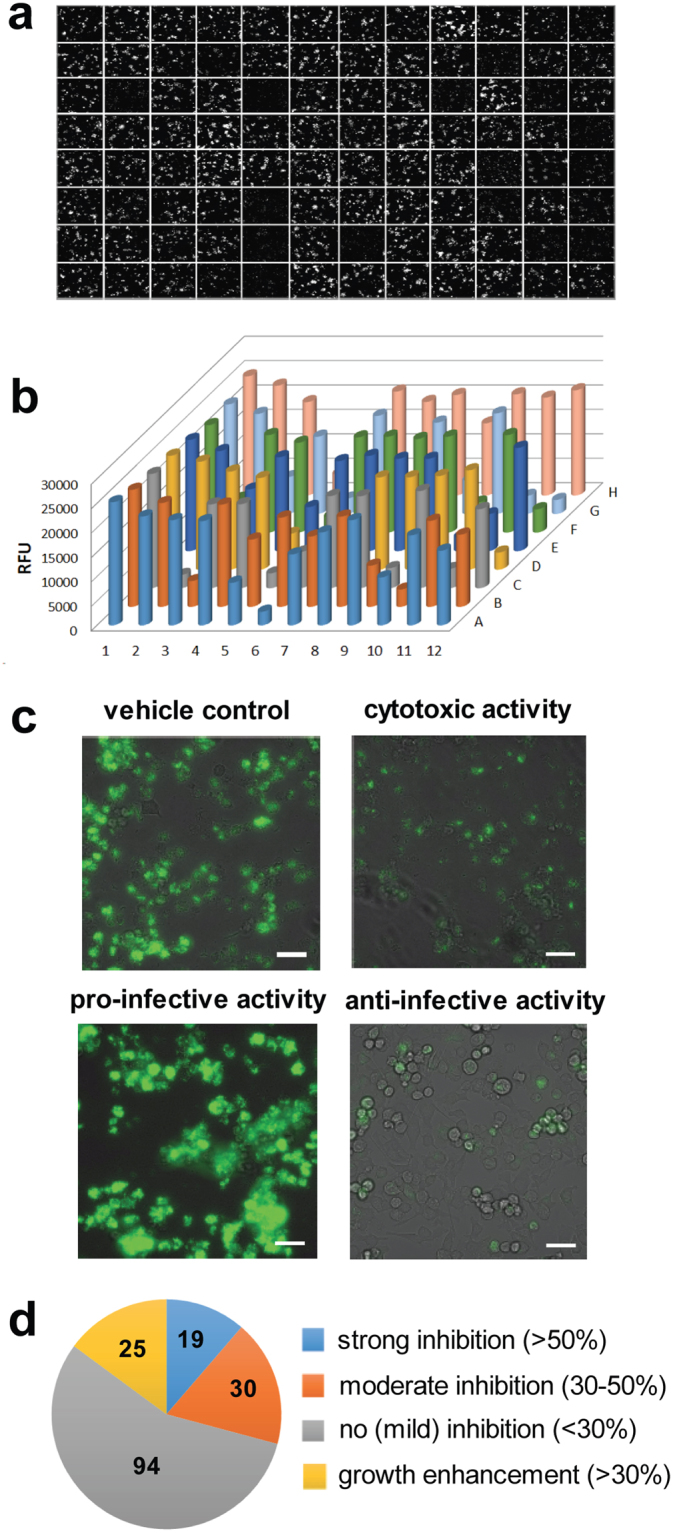
Characterization of anti-infective activities of GSK TB-set compounds in a mammalian infection assay. (**a**) Confocal microscopy of 10^5^ *BV2* microglial cells infected with *M. marinum* msp12::GFP, 60 hours incubation time in DMEM medium supplemented with bovine serum in a 96-well plate. Compounds derived from GSK TB-set were added at 10 μM dissolved in DMSO. (**b**) 3D-chart representing quantification of fluorescence in GSK TB-set. (**c**) Confocal/brightfield microscopy of BV2 cells infected with *M. marinum* msp12::GFP in the presence of vehicle control (B), cytotoxic (C), anti-infective (D) and pro-infective (E) compounds. 10^5^ BV2 cells were transferred to the wells. (**d**) Pie chart representing summarized results of growth inhibitory activity of 168 compounds in *A. castellanii* infection assay. The analysed set included compounds that displayed inhibition of more than 50% normalized RFU (blue), moderate inhibition between 30 and 50% (red), compounds that showed less than 30% inhibition (grey) and compounds that displayed increase of RFU by more than 30% (yellow). The scale bar corresponds to 500 µm.

We established an infection assay based on the BV2 microglial cells and *M. marinum* expressing GFP to measure the anti-infective activity of compounds from the GSK TB set. The assay was designed to be as similar as possible to the *A. castellanii* assay for the ease of results comparison. For this, BV2 cells were infected with GFP-expressing *M. marinum* and intracellular bacterial growth was monitored for three days in the presence of 10 μM amikacin. For quantification of *M. marinum* growth, we used time-lapse imaging with a high-content fluorescence microscope (Fig. 4A–D,F). Results of two independent experiments are presented in Tables 1 and S1 and microscopy pictures are presented in Figure S7. Only 19 compounds (11.3%) were found to possess strong anti-infective activity, with more than 50% inhibition of bacteria growth (Figure S6A). Thirty showed moderate inhibition between 30 and 50%. Interestingly, 25 compounds (14.9%) also demonstrated clear pro-infective activity, resulting in more than 30% increase of bacterial growth during the infection in the presence of the vehicle control (Fig. 4G). Together, the results from both the *A. castellanii*- and BV2-based assays indicate a significant decrease in antibacterial activity of most compounds in comparison to the antibiotic assay in broth. These high attrition rates correspond in magnitude to the ones observed in most anti-tubercular screens, highlighting the biological significance and relevance of our screening systems^13^. Interestingly, we observe similar numbers of hits using the two infection systems, and the strongest hits are the same in the BV2 and *A. castellanii* based assays (see Tables 1 and S1, coloured in orange and dark red, respectively). In the case of the BV2/*M. marinum* infection assay, the IC50 of each compound was determined by high-content microscopy (Table S1, Figures S7, S8).

### Amikacin does not significantly interfere with the detection of anti-infective hits against *M. marinum* during infection of BV2 cells

To determine whether amikacin affects the intracellular growth of *M. marinum* during infection, independently of, or in synergy with, the tested compounds. Amikacin is a crucial element in our infection assays, because it is continuously present in the medium at 10 μM. Amikacin has low cellular permeability^15^ and should not interfere with intracellular growth. The presence of amikacin in the medium during the course of the assay, and not only right after spinoculation, is important since we detect events of bacterial escape from the amoeba host^36^ that can lead to extracellular growth. However, our BV2 cell-based infection assay demonstrated insignificant numbers of bacterial escape (Video 1). This property of the BV2 host allowed us to compare the influence of amikacin on infection. We analysed the hit patterns emerging in the BV2 infection assay (described above) in the presence or absence of 10 μM amikacin, and found that amikacin does not influence hit identification. However, it should be noted that amikacin did affect the general fluorescence intensity of the bacterial load (Figure S7). Our results indicate that amikacin has an effect on mycobacteria growth inside BV2 cells, however, this effect did not significantly change the outcome of the screen.

### Determination of antibacterial potency and structure-activity relationships of a set of 18 compounds against *M. marinum* during infection of *A. castellanii*

The relative abundance of chemical analogs within the GSK TB-set allowed us to perform a limited set of structure-activity relationship studies in the case of the imidazo[1,2-a]pyridine-3-carboxamide family of compounds, represented with 18 compounds in the set analysed (Figures S9, S10). This family showed high anti-tubercular potency both in *A. castellanii* and BV2-based assays. Chemical similarity of compounds was estimated by Tanimoto coefficient and analysis of substructures. This family and its antitubercular activity was already mentioned in the literature^41^. Compounds were tested at 0.01, 0.1, 1, 2, 5, 10 and 20 µM in the *A. castellanii*/*M. marinum* system. MarivlSketch was used to visualize the compounds at atomic level. The GraphPad Prism software was used to generate MIC curves. Canvas with the Schrodinger package is used for calculation of nonlinear Kernel-based partial least squares regression (KPLS). The nonlinear regression was based on the data of 12 compounds with IC_50_ less than 20 µM. The KPLS regression model shows coefficient of similarly Q^2^ r0.0728 and regression coefficient R^2^ 0.8043. The relatively low coefficient of predictability can be explained by the small size of the set of analysed compounds and low numbers of biological replicates. A significant increase in the number of chemical analogs will be required to construct more precise models of structure-activity interactions.

### Identification of QcrB and KasA as molecular targets of GSK1829820A and GSK3011724A respectively and DHFR as a target of 30 small molecules of the GSK TB-set

A number of other compounds listed in the table have had their mode of action delineated in previous publications. The molecules that have been annotated as targeting DHFR (mycobacterial dihydrofolate reductase) were identified by *in silico* docking assay^42^. Spontaneous resistant mutants of *M. bovis* BCG were generated on solid mycobacterial media at 5 times the MIC of the corresponding compound. Following the sequencing of the mutants and molecular genetic validation of the mode of action, the imidazo[1,2-a]pyridine compounds were found to target the *b* subunit of the mycobacterial cytochrome *bc*_1_ complex (QcrB). One of these compounds, GSK1829820A, has been identified in a high-content screen^43^. This method of mode of action elucidation by resistant mutant generation also previously provided the target of GSK3011724A, as inhibiting the essential long-chain fatty acid synthase enzyme β-ketoacyl synthase (kas) A^44^.

### Identification of MurC as the target of GSK831784A and GSK847920A, and FbpA as the target of GW859039X

**Figure 5:**
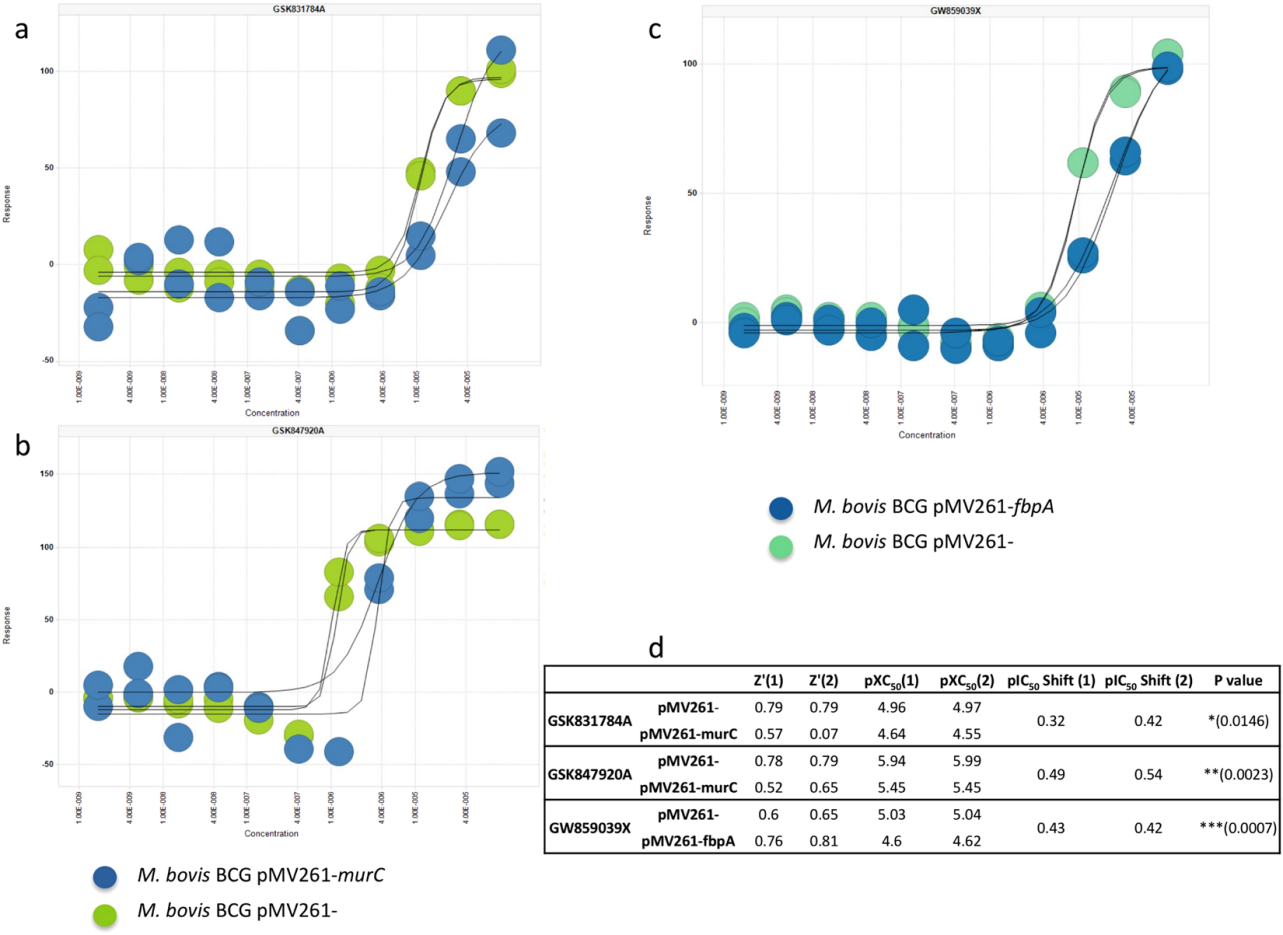
Identification of molecular targets of compounds from GSK TB-set. The shift in cell viability against a dose response of (**a**) GSK831784A and (**b**) GSK847920A upon over expression of MurC. Over expression of FbpA led to a shift in dose response to (**c**) GW859039X. (**d**) A tabulated summary of pXC50 shift values of each strain against the respective compounds.

The mode of action of these new anti-mycobacterial compounds was discovered using a target-specific high-throughput screening technology, following a previously described method^45^ using *M. bovis* BCG over-expressing the essential mycobacterial genes *murC* (*Rv2152c*) and *fbpA* (*Rv3804c*), encoding the UDP-N-acetylmuramate-alanine ligase (MurC) and mycolyl transferase 85 fibronectin-binding protein A (Antigen 85 A), respectively. These overexpressing strains were used to probe the hit compounds identified in the *A. castellanii -M. marinum* infection high-throughput screen that had not previously had their mode of action delineated^40^. A commercially available luciferase reporter assay was used to measure cell viability of each strain following 7 days of incubation with each of the compounds at 1 and 10 μM in a 1536-well format. Hits were identified based on shift in apparent inhibition (calculated as % inhibition of *M. bovis* BCG pMV261 (empty plasmid) minus % inhibition of *M. bovis* BCG pMV261-*murC/fbpA* [based upon duplicate data]). Hit compounds were analysed using a serial dilution from 10 mM to 0.17 nM against *M. bovis* BCG pMV261 and *M. bovis* BCG pMV261- *murC/fbpA*. The percentage inhibition at each concentration was used to determine the XC_50_ (compound concentration required to inhibit cell viability by 50%) of each compound against the over-expresser and empty vector strains based on duplicate data. This resulted in the identification of the mode of action of three of the compounds by XC_50_ shift upon target over-expression. The XC_50_ data for *M. bovis* BCG pMV261-*murC* against GSK831784A and GSK847920A, and *M. bovis* BCG pMV261-*fbpA* against GW859039 × along with the respective empty vector controls is shown in Fig. 5.

### Identification of MmpL3 and GlpK as the targets of GSK2043267A and GSK1742694A, respectively

Remaining hit compounds lacking mechanism of action delineation were entered into a pipeline of spontaneous resistant mutant generation, wherein resistant isolates were selected from 10^8^ colony forming units of *M. bovis* BCG against compounds at high concentrations with respect to the minimal inhibitory concentration (MIC). Spontaneous resistant mutants were generated at 5 times the established MIC. Four resistant isolates were identified for GSK1742694A, and ten resistant isolates were identified for GSK2043267A. Phenotypic resistance was confirmed by plating resistant isolates at 0 and 5 times MIC alongside wild type *M. bovis* BCG (Figure S11). Upon confirmation of the resistance phenotype, the genomic DNA was isolated and sequenced (MicrobesNG) by Illumina sequencing. A resistance-conferring single nucleotide polymorphism (SNP) was identified in all ten of the resistant isolates raised against GSK2043267A, in the Mb0212c gene at position F255L (Ttc/Ctc) encoding MmpL3. Sequencing of the four mutants raised against GSK1742694A yielded a high-frequency SNP (ttG to ttC) corresponding to L385F in Mb3721c, encoding the glycerol kinase GlpK. Both of these genes have predicted essentiality in *Mtb* H37Rv^46^.

## Discussion

In our study, we characterized 168 compounds from the GlaxoSmithKline TB-set *in vivo*, using protozoan and mammalian hosts for the infection with *M. marinum* as a model of *Mtb*. These chemicals were derived from an original GSK collection of 2 million compounds and display high antibiotic activities against *M. bovis* BCG and *Mtb* H37Rv, and low or no cytotoxicity in a HepG2 assay. We selected the murine microglial BV2 cells as a model of mammalian macrophage. The choice of protozoan hosts included the free-living amoebae *A. castellanii* and *D. discoideum*. Constitutive expression of GFP-reporters was used as the main readout for the quantification of prokaryotic and eukaryotic cell numbers. The methodology of the screening has already been established and validated with known antitubercular compounds in our lab^37^.

Here, we characterized the compounds in five different screens, including assays to monitor the intracellular growth of *M. marinum in A. castellanii* and BV2 microglial cells. We performed time-resolved measurements that are far superior and richer in information about the impact and mode of action of the compounds than the traditional end-point measurements. First, we detected the growth of *M. marinum* in Middlebrook 7H9 medium in the presence of compounds. The results of the screen are presented in Tables 1 and S1. The data revealed that 122 compounds (72.0%) displayed significant inhibitory activity against *M. marinum*-GFP at a 10 μM concentration. The remaining 28.0% of the hits are probably not detected due to genetic and host-range differences between *Mtb* and *M. marinum* and possibly some technical factors. The use of an auto-bioluminescent *M. marinum* strain corroborated the data obtained with the GFP-fluorescent strain, but showed generally higher levels of inhibition of bacterial growth resulting in 94% of compounds displaying notable inhibition. The difference between the two assays most likely corresponds to the nature of the two readouts. While the bacterial luciferase-based assay depends on the bacteria ATP level and thus monitors both the total bacterial mass and metabolic status, the GFP-signal corresponds in first approximation to the bacterial mass of both live and dead mycobacteria. Indeed, it is known that the fluorescence does not go off immediately when bacteria are killed^47^. The choice between luminescence- and fluorescence-based readouts remains subjected to the balance between their intrinsic advantages and limitations.

Overall, the antibiotic activity screen against *M. marinum* in broth displayed a substantial overlap with the results of the original screen against *Mtb H37Rv*^40^, despite the significantly shorter duration of the assay. This may speak in favour of *M. marinum* as a substitute for *Mtb* in antibiotic screening, given its ease of manipulation and cultivation, lower health and safety risks (biosafety level 2) and a fast growth rate that decreases the duration of experimental procedures. Additionally, because *M. marinum* is a fully active pathogen, unlike *M. smegmatis* or even *M. bovis* BCG, which are often used for compound screening, it offers the possibility to transition from single-cell assays to vertebrate organisms by using the powerful zebrafish infection system. In recent years, this system has become widely recognised as a strong alternative model to study the pathogenesis of TB^40,48^.

Estimation of the compounds’ cytotoxicity/growth inhibition activity was performed by monitoring the growth of GFP-ABD *D. discoideum* and BV2 cells. As a confirmation and expansion of the cytotoxicity tests performed in Ballel *et al*.^40^, only low numbers of cytotoxic activities were identified, 2.3% for *D. discoideum* and 3.2% for BV2, with 3 compound having cytotoxic activities in both assays. We observed a wider range of growth inhibitory activities in the *D. discoideum* assay. The observation of compounds in the TB-set showing cytotoxicity towards microglial cells seems surprising because they passed a standard HepG2 cytotoxicity assay^40^. Additionally, the *A. castellanii*-based growth inhibition assay provides results similar to the *D. discoideum*-based growth inhibition assay, confirming overall conservation of basic biological and metabolic pathways. In summary, compared to the original test performed on HepG2 cells^40^, *D. discoideum* is just as predictive of cytotoxic compounds as any other mammalian cell line such as BV2. Given the overall advantages of *D. discodeium*, including the ease of genetic manipulation, this system generates new opportunities to quantitate cytotoxicity in any drug development process.

Our study reports a parallel and extensive comparison of the intracellular activity of anti-tubercular compounds in both a protozoan model infection system (*A. castellanii*), and a mammalian macrophage cell line (microglial BV2 cells). We revealed a significant attrition of the antibacterial activity both in the *A. castellanii* and the BV2 infection systems. We detected 8 hits (4.8%) that inhibited more than 50% of the intracellular growth of *M. marinum* at 10 μM in *A. castellanii* and 19 hits (11.3%) in the BV2 microglial cells. We also identified 24 and 30 moderate anti-infective hits with inhibition of 30 to 50% in the *A. castellanii* and BV2 cells, respectively. The inability of reaching 100% inhibition in most of the cases can be expected given the short duration of the assays (60–72 h) and stability of GFP-reporters used for mycobacteria quantification. Our data suggest that an incomplete yet reproducible inhibition might be sufficient for the detection of antibacterial hits. The higher stringency of the amoeba-based assay was expected because amoebae live in chemically challenging environments and are known to possess a large range of export pumps for xenobiotics^49^. However, the overall similar levels of attrition indicate that the evolutionary origin of the host cell, e.g. protozoan versus mammalian, is not the dominant factor in anti-infective hit identification. Instead, we suggest that the technical aspects of the screen may play an important role, including factors such as media composition, temperature, multiplicity of infection and others. The presence of amikacin in the medium might be one of these factors. In the BV2 infection assay, however, we did not find noticeable synergistic activity of amikacin with other compounds. Nevertheless, decreased bacteria growth indicated that amikacin exerts a significant effect on intracellular bacteria, despite previous reports^15^.

A pronounced decrease in the number of compounds exerting a notable intracellular antibacterial activity is not so surprising and corresponds to the well-known high attrition rate in antibacterial drug discovery, for which, according to industry averages, the success rate is about 25% at early steps, while the complete process takes an astonishing 13 to 14 years^13^. In this regard, the *A. castellanii* infection assay revealed results comparable to the microglial cell infection assay (see below) in terms of hit numbers. Indeed, four of the identified hits bear strong anti-infective properties in both assays including compounds GSK3011724A, GSK1985270A, GSK1055950A, GSK353069A. Among moderately anti-infective compounds, 21 were identified both in the amoeba and microglia-based assays. All the hits identified in infection assays showed strong antibiotic activities against *M. marinum* in bacterial broth. Our results show the *A. castellanii*-based assays to be predictive for activity in mammalian cells, although the amoeba assay is more stringent. This property may be beneficial for excluding inconclusive hits during hit-to-lead drug development.

**Figure 6:**
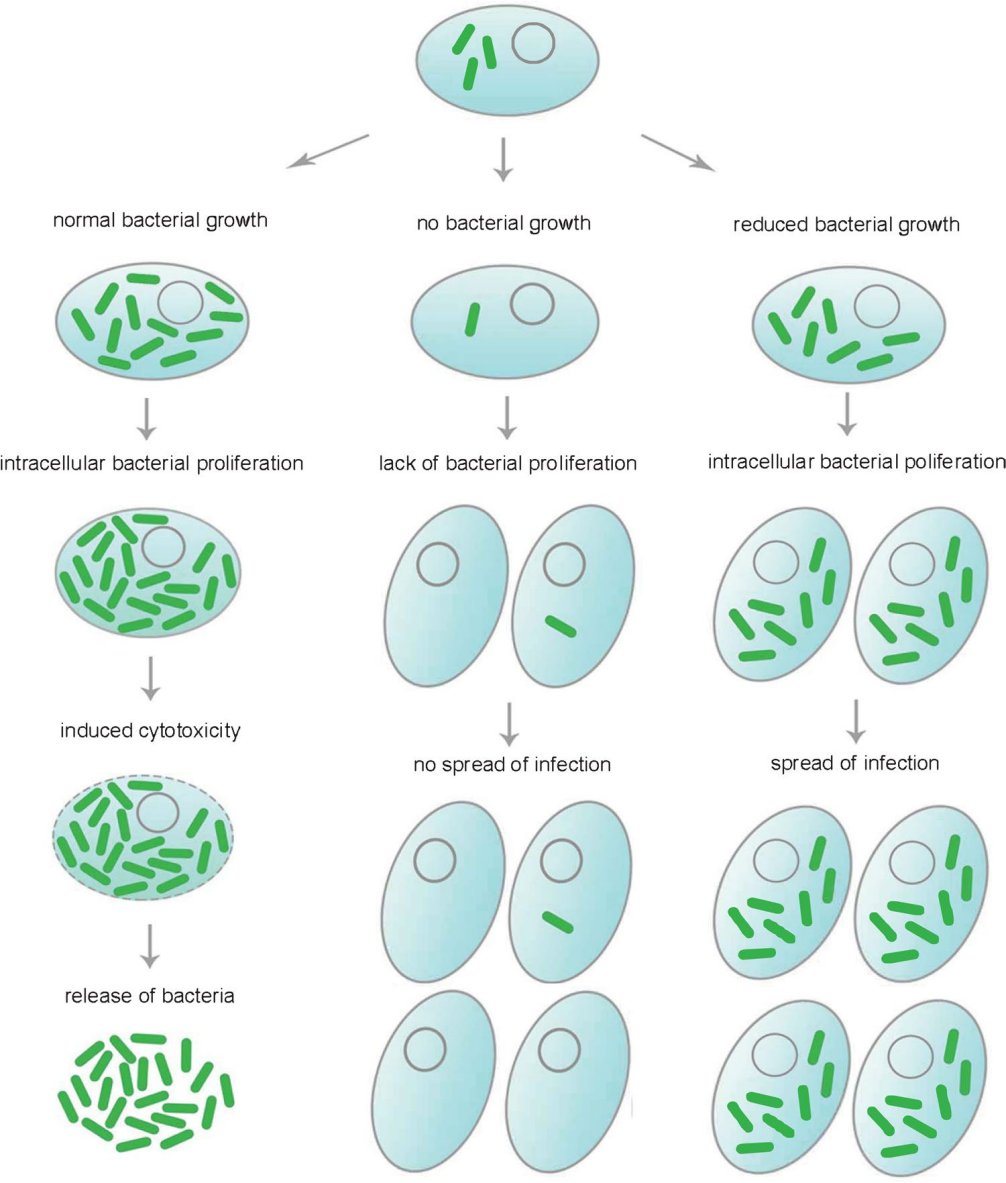
Scenario for the pro-infective activity of some antibiotics. A compound with strong antibiotic activity simply kills bacteria inside the host and cures the infection, resulting in a rapid leveling of the total fluorescence intensity of GFP-expressing *M. marinum*. A compound that lacks anti-infective activity does not prevent intracellular mycobacterial growth, causing early proliferation of the bacteria and the death of the host. The bacteria kill their growth niche and thus loose access to host resources, which together with the extracellular activity of amikacin limits the maximal fluorescence increase. Counterintuitively, a compound with mild anti-infective activity will decrease the fitness and virulence of intracellular *M. marinum*, thereby limiting its cytotoxicity towards the host cell. The bacteria thus proliferate, use cellular resources and spread the infection without killing the host, reaching potentially higher maximal fluorescence.

On the other hand, identification of infection enhancers in the set of characterized antibiotic compounds was quite unexpected and has not been previously reported. Notably, we identified a certain number of pro-infective compounds, namely chemicals that increase the overall bacterial mass of *M. marinum* using both the BV2 and *A. castellanii* infection assays. 13% of the compounds demonstrated pro-infective activity in the *A. castellanii* infection assay and 14% in the BV2 cells infection assay. This type of activity may usually be largely ignored in TB drug screening campaigns. At the same time, such compounds may be beneficial for the understanding of drug action, because they may unveil important signalling pathways important for boosting anti-bacterial host defences. Intriguingly, the majority of identified pro-infective compounds were different in amoeba and mammalian cells-based assay (with an overlap of only 3 compounds), which indicates lower levels of conservation of pro-infective mechanisms compared to anti-infective activities. It must also be emphasized that the strongest pro-infective compounds (more than 70% increase) in the amoeba infection assay belong to the same chemical family (Table S1) and exclusively build-up this pro-infective class. We suggest a model that explains such activities (Fig. 6). We propose three main scenarios for the bacteria fates in the presence of the compound. In all three cases, it is important to note that the fluorescence intensity is proportional to bacterial mass, but bacteria remain fluorescent even when they die. The first scenario illustrates the standard bactericidal/bacteriostatic activities that result in a decrease of bacteria growth rates, as predicted. The second scenario takes into account the absence of significant bactericidal effect. In this case, the output is similar to an infection in the presence of the vehicle control, in which bacteria proliferate intracellularly and cause death of the host. The extracellular amikacin then restricts the bacterial mass. The third scenario represent the effect of compounds that induce a slight weakening of intracellular mycobacteria, lowering their cytotoxicity for the host cells. The access to the host resources and the shielding property of the host cell are therefore dominant factors, which lead to a relatively increased total mass of *M. marinum* compared to control conditions.

We identified four new molecular targets for compounds from the GSK TB-set. MmpL3 and GlpK are the targets of GSK2043267A and GSK1742694A, respectively. MurC is proposed as the target of GSK831784A and GSK847920A, and FbpA as the target of GW859039X. FbpA, a diacylglycerol acyltransferase/mycolyltransferase, synthesizes trehalose 6,6′-dimycolate, the key component of outer envelope in *Mtb* H37Rv. Mycolic acids of trehalose dimycolate also interact with the mycolyl-residues from the layer beneath. FbpA is a component of the antigen 85 (Ag85) complex, which together with FbpB and FbpC. FbpA, has been shown to be crucial for the growth in nutrient-poor media and a macrophage-like cell line^50^. FbpA might play a crucial role in both cell wall stability and the biosynthesis of storage compounds for the survival of *Mtb* in the non-replicating persistent state during which mycobacteria use fatty acids as a carbon source. Another identified target, MurC is a member of Mur ligases, the key enzymes of the peptidoglycan biosynthesis pathway. Like fatty acids, peptidoglycans are essential components of the cell wall that provide a rigid support maintaining cell shape and turgidity. Mur synthetases are key enzymes in the cytoplasmic steps of PG biosynthesis. MurC initiates the synthesis of the stem peptide by addition of L-alanine (L-Ala) to the carboxyl group of uridine-diphospho-N-acetyl-muramic acid (UDP-MurNAc). It was shown that MurC was able to incorporate glycine (Gly) and L-Ala to UDP-MurNAc in both *Mtb* and *M. leprae*^51^. Munshi and colleagues (2013) observed that MurC synthetase from *Mtb* showed additional activity with L-Ser as confirmed by HPLC and LC-MS^52^. Fatty acid biosynthesis was also displayed by identification of MmpL3, essential mycolic acid transporter, as a target. Indeed, the inhibition of MmpL3 results in the abolition of the export of mycolic acids to the outer membrane and in the rapid killing of the bacilli^53,54^. There are 4 inhibitors already identified, displaying promising results based on efficacy, tolerability and pharmacological properties^55–58^. The high potency of these compounds displays vulnerability of the MmpL3 transporter both *in vitro* and *in vivo*^53^. GlpK is the key enzyme in the regulation of glycerol uptake and metabolism that catalyses the phosphorylation of glycerol to yield sn-glycerol 3-phosphate.

Only one of the compounds with identified molecular targets, GSK1742694A, displayed low antibiotic activity in our *M. marinum* growth assay. This is probably due to the short duration of the assay that prevents bacteria from exploiting their glycerol metabolism. However, GlpK could be a hit with no real physiological relevance, because our antibiotic screen as most whole-cell high throughput screens was performed with medium containing glycerol as the main carbon source, which raises a possibility of GlpK identification being a side effect of the selected experimental methodology. Example of such false positive hits are exemplified in a published study^59^. On the contrary, GSK2043267A, GSK831784A, GSK847920A, GW859039X displayed strong antibiotic activity in the *M. marinum* growth assay and variable activity in infection assays (Table S1).

Recently, Av-Gay and colleagues characterized 144 compounds of the GSK TB-set in Mtb-H37Rv-infected THP1 cells, using a luciferase reporter for bacterial quantification^60^ (Table S1). That assay displayed generally higher level of antibiotic activity, with 90.3% of the compounds confirmed as active in the intracellular environment. The present study bears some similarities in scope and experimental strategy with that of Sorrentino *et al*.^58^, however it must be noted that the study of Av-Gay *et al*. uses *Mtb* instead of *M. marinum*, and of a macrophage host cell line that is widely used in the field, compared to a microglial cell line. On the other hand, the alternative amoeba host systems used in our study are intrinsically more stringent in terms of revealing intracellular anti-infective molecules^37–39^, and because of the evolutionary distance automatically selects “universally active” compounds. In addition, while they used mainly an end-point measurement, we performed time-resolved measurements that are superior and richer in information about the impact and mode of action of the compounds. Some other differences with our results may be caused by technical factors, specifically higher loads of intracellular bacteria in our assays (Video 1); the use of a luciferase versus a fluorescence reporter. Despite these differences, among the 10 most potent compounds identified^60^, 8 were also very potent in our luciferase-based assay. These compounds displayed variable activities in BV2 or *A. castellanii* infections, with GSK1985270A having the most prominent antibacterial effect. Interestingly, we identified MmpL3 as a target of GSK1589673A, but have not identified resistant mutants in the case of GW623128X, GSK1589673A, GSK937733A GSK1589671A, compounds that are presumed to target MmpL3^60^. Notably, 3 out of 10 compounds displayed pro-infective activity in our BV2 infection assay. Such an apparent reversal between anti- and pro-infective activity was observed for a few of the compounds when switching between the cellular models used for infection (see Table S1). This phenomenon might be explained by the three scenarios presented in Fig. 6.

The present study represents a welcome diversification of the strategies for anti-TB drug discovery, which is important to increase the success rate of anti-TB screening campaigns. In particular, four new molecular targets have been identified. Therefore, because of the conservation of mechanism of action between *M. marinum* and *Mtb* as well as the fact that the compounds have anti-infective activities in multiple eukaryotic host systems, these molecules may have significant therapeutic potential. We expect that our effort with the use of classical and alternative whole cell-based TB screening for characterization of the GSK TB-set can support the fight against TB. Identification of drugs targeting cell wall stability and biosynthesis is an important step towards the elimination of the TB threat.

## Materials and methods

### Strains, transformation and culture

For *M. marinum*-based experiments the M strain was stably transformed with the pMV306-luxCDABE expression plasmid^61^, rendering them auto-bioluminescent, or with the msp12::GFP expression vector (from Dr. L. Ramakrishnan)^62^ to obtain green-fluorescent bacteria. The mycobacteria strains described are available from the authors. For plasmid-carrying *M. marinum* strains, the growth medium was supplemented with 25 μg /ml kanamycin. Experiments for molecular target identification were performed in *M. bovis* BCG that has been transformed by a standard heat shock protocol with pMV261, a kanamycin-resistant mycobacterial over-expresser plasmid, which had been modified by addition of either Mtb *murC* (Rv2152c) or Mtb *fbpA* (Rv3804c). An empty vector strain (pMV261 only) was also prepared. Transformants were selected on Middlebrook 7H11 mycobacterial media (Difco) supplemented with 10% (v/v) OADC (oleic acid (1.25 × 10–2% v/v), albumin (1.25% w/v), dextrose (0.5% w/v) and catalase (1 × 10–3% w/v) (Sigma-Aldrich)), containing kanamycin at 25 μg.ml-1. Successful transformants were transferred into Middlebrook 7H9 liquid media (Difco) containing kanamycin at 25 μg.ml^−1^, 0.05% (v/v) Tween-80, 0.25% (v/v) glycerol and supplemented with 10% (v/v) ADC (albumin (1.25% w/v), dextrose (0.5% w/v) and catalase (7.5 × 10–4% w/v) (Sigma-Aldrich)). Growth rate was monitored by measuring optical density (OD) at 600 nm and cells were passaged to maintain a mid-log OD_600_ of 0.4–0.8. To prevent mutagenesis, no more than 4 passages were conducted before cultures were reinitiated from a glycerol stock. In eukaryotic host-based assays *A. castellanii* (ATCC 30234)^63^ and murine microglial BV2 cells^64^ were utilized.

### Antibiotic activity assay (*M. marinum*)

GFP-expressing or luxCDABE-expressing *M. marinum*^65^ were cultivated in a shaking culture at 32 °C up to an OD600 of 0.8–1 in 7H9 medium supplemented with OADC. 10^5^ GFP-expressing *M. marinum*^65^ were transferred into each well of 96-well white plates. Bacterial growth at 32 °C was monitored by measuring the fluorescence, OD or luminescence in a plate reader (Synergy H1) for at least 48 hours with a time point taken every 3 hours^66^ and by CFU counting. Rifabutin was added as a control at 10 µM concentration. Fluorescence excitation was at 485 nm and emission was monitored at 509 nm and light scattering measurements were performed at 600-nm. For CFU counting, samples were withdrawn and then serially diluted in saline solution (PBS). Aliquots of 100 μl were spread on 7H10 agar plates. After incubation at 32 °C, colonies were counted to determine the number of CFU.

### Growth assay (*D. discoideum*)

10^4^ GFP-ABD-expressing *D. discoideum* cells were transferred into each well of 96-well plates (Cell Carrier, black, transparent bottom from Perkin-Elmer), and allowed to attach for 20–30 min. Cell growth at 25 °C was monitored by measuring the GFP fluorescence in a fluorescent plate reader (Synergy H1, company) for at least 48 hours with a time point taken every 3 hours.

### *A. castellanii* infection assay

*A. castellanii* were cultured in PYG medium in 10 cm Petri dishes at 25 °C, and passaged the day prior to infection to reach 90% confluency. *M. marinum* were cultivated in a shaking culture at 32 °C to an OD_600_ of 0.8–1 in 7H9 medium. Mycobacteria were centrifuged onto a monolayer of *A. castellanii* cells at an MOI of 10 to promote efficient and synchronous uptake. Centrifugation was performed at RT at 500 g for two periods of 10 min. After an additional 20–30 min incubation, extracellular bacteria were washed off with PYG and infected cells were resuspended in PYG containing 10 μM amikacin. 5 × 10^4^ infected cells were transferred to each well of a 96-well plate (Cell Carrier, black, transparent bottom from Perkin-Elmer) with preplated compounds and DMSO/rifabutin controls. The course of infection at 25 °C was monitored by measuring fluorescence in a plate reader (Synergy H1, BioTek) for 72 hours with time points taken every 3 hours. Time courses were plotted and data from all time points were used to determine the effect of compounds versus vehicle controls. To take into account possible autofluorescence of the compounds, RFU data of the first time point were subtracted from all time points. Cumulative curves were calculated. The activities of the compounds were determined by analysing maximum difference of compound cumulative curve to the 12–16 vehicle controls. For CFU counting, samples were withdrawn and then serially diluted in saline solution (PBS). Aliquots of 100 μl were spread on 7H10 agar plates. After incubation at 32 °C, colonies on the plates were counted to determine the number of CFU.

### BV2 cells infection assay

BV2 cells were cultured in DMEM medium supplemented with FCS in 10 cm Petri dishes at 37 °C. The day prior to infection, cells were trypsinized, passaged and transferred to 96-well plates (black transparent bottom from BD Falcon), so as to reach 60–70% confluency the day of the experiment. *M. marinum* were cultivated in a shaking culture at 32 °C up to an OD600 of 0.8–1 in 7H9 medium supplemented with OADC. Mycobacteria were centrifuged onto preplated BV2 cells at an MOI of 3 to promote efficient and synchronous uptake. Centrifugation was performed at RT at 500 g for 10 min in a clinical centrifuge. After an additional 20 min incubation, extracellular bacteria were washed off with DMEM and infected cells were resuspended in DMEM supplemented with FCS. Compounds from the GSK TB set were added to a 30 µM final concentration. Infected cells were incubated at 32 °C. The course of infection was monitored by high-content microscopy with time points taken every 7–14 hours.

### High Content microscopy

Infected cells were monitored in 96-well plates (black, transparent bottom from BD Falcon). Recording of transmitted light and GFP fluorescence data were performed using ImageXpress Micro XL Widefield High-content Screening System (20 × 0.75 NA, air). Quantification of the image fluorescence intensity was used to determine the effect of compounds versus vehicle controls. The activities of the compounds were determined by analysis of the maximum difference of fluorescence intensity at day 3 post-infection compared to 6–16 vehicle controls. High Content microscopy were used for conformation of IC_50_ data generated by single point measuring fluorescence in a plate reader (Synergy H1, BioTek).

### Qualitative SAR studies

Compounds of interest were assayed at a range from 0.01 to 20 µM concentration in *A. castellanii – M. marinum* model, last data points values at 3 days post-infection were used to build dose response curve in Graph Pad Prism to determine IC_50_. A mean value from at least two biological replicates is given for each compounds.

### High-throughput screening *of* hit compounds using *murC* and *fbpA* over-expressing strains of *M. bovis* BCG

Hit compounds with activity against *M. marinum-*infected *D. discoideum* identified in the high-throughput screen were tested for mechanism of action using *murC* and *fbpA* over-expressing strains of *M. bovis* BCG following the same methodology as previously described^45^. This was first conducted in single shot format at 10 and 1 μM, followed by a dose response validation of in serial dilution of 11 steps from 10 mM to 0.17 nM using a 1/3 dilution factor all in a 50 nl total volume with relevant controls. All data regarding XC_50_ shift was obtained in duplicate.

### *M. bovis* BCG spontaneous *mutant* generation and sequencing

The MIC of each compound against *M. bovis* BCG was determined on solid (Middlebrook 7H11) mycobacterial media and resistant mutants generated at 5 times the MIC. Resistant mutants were verified by plating back onto selective media alongside wild type before genomic DNA extraction and whole genome sequencing. The methodology herein is as described in Abrahams *et al*. 2012^43^.

### Data availability

Most data generated or analysed during this study are included in this published article (and its Supplementary Information files). Additional datasets generated and analysed during the current study are available from the corresponding authors on reasonable request.

## Electronic supplementary material

Supplementary information

Video 1

Video 2

Video 3

Table S1
