# Supplementary material for: Antimycobacterial drug discovery using Mycobacteria-infected amoebae identifies anti-infectives and new molecular targets
Source: Sci Rep. 2018 Mar 2;8:3939. doi: 10.1038/s41598-018-22228-6 (PMC5834492; doi:10.1038/s41598-018-22228-6)
Supplement: Supplementary file 1 — Supplementary information [file 41598_2018_22228_MOESM1_ESM.docx]

**Supplementary informations**

**Antimycobacterial drug discovery using Mycobacteria-infected amoebae identifies anti-infectives and new molecular targets**

Valentin Trofimov^1, 2^, Sébastien Kicka^1^, Sabrina Mucaria^3^, Nabil Hanna^1^, Fernando Ramon-Olayo^4^, Laura Vela-Gonzalez Del Peral^4^, Joël Lelièvre^4^, Lluís Ballell^4^, Leonardo Scapozza^3^, Gurdyal S. Besra^5^, Jonathan A.G. Cox^6, *^ and Thierry Soldati^1, *^

^1^Department of Biochemistry, Faculty of Science, University of Geneva, Geneva, Switzerland

^2^Present address: Institut Pasteur de Lille, Lille, France

^3^Pharmaceutical Biochemistry/Chemistry, School of Pharmaceutical Sciences, University of Geneva, Geneva, Switzerland

^4^GSK, Severo Ochoa 2, 28760 Tres Cantos, Madrid, Spain

^5^School of Biosciences, University of Birmingham, Edgbaston, Birmingham, UK

^6^School of Life & Health Sciences, Aston University, Birmingham, UK

^*^Co-corresponding authors:

Dr. Thierry Soldati

Department of Biochemistry

Faculty of Science

University of Geneva

30 quai Ernest Ansermet, Sciences II

CH-1211-Genève-4, Switzerland

**Tel**: [+41-22-379-6496](tel:%2B41-22-379-6496)

**Email:** [Thierry.Soldati@unige.ch](mailto:Thierry.Soldati@unige.ch)

Dr. Jonathan A. G. Cox,

School of Life & Health Sciences

Aston University

Aston Triangle

Birmingham B4 7ET UK

**Tel**: +44 121 204 5011

**Email**: [j.a.g.cox@aston.ac.uk](mailto:j.a.g.cox@aston.ac.uk)

**Supplementary table, figures and videos legends**

**Table S1. Summary of the activities detected by screening the GSK TB-set**. The antibiotic activity of the compounds was monitored using fluorescent GFP-expressing or auto-bioluminescent luxCDABE-expressing *M. marinum.* Growth inhibition and cytotoxicity were measured on GFP-ABD expressing *D. discoideum* (in quadruplicate) and BV2 microglial cells (single endpoint measurement). Infections with *M. marinum* were performed in *A. castellanii* and BV2 microglial cells. The screenings were performed for 72 hours. Mean values of fold increase are presented; data is normalized to vehicle control. Corresponding standard deviations are indicated. The IC50 of the compounds in the BV2/*M. marinum* infection assay was calculated. The identification of molecular targets was based on generation of over-expresser *M. bovis* BCG strains, generation of spontaneous resistant mutants and whole genome sequencing.

**Figure S1. Fluorescence cumulative curves of compounds of TB-set in *M. marinum* screen in 7H9 broth**. Plates GSK1 (A) and GSK2 and GSK3 (B). 10^5^ *M. marinum* expressing GFP were transferred to the wells. Compounds derived from GSK TB-set were added at 10 μM dissolved in DMSO. Fluorescence intensities were measured for 48/72 hours every 3 hours. Reproducible characteristics of *M. marinum* growth allowed decreasing of the measurement duration from 72 down to 35-42 hours.

**Figure S2. BV2 growth inhibition assay.** 5x10^4^ infected BV2 cells were transferred to the wells, in DMEM medium supplemented with fetal bovine serum. Either vehicle control (green frame) or the compounds derived from GSK TB-set were added at 10 μM concentrations. Measurement is taken 50 hours post infection.

**Figure S3. Fluorescence cumulative curves of compounds of TB-set in *D. discoideum*-GFPABD screen in HL5c medium.** Plates GSK1 (A) and GSK2 and GSK3 (B). 10^5^ *D. discoideum* cells expressing GFP-ABD were transferred to the wells. GSK TB-set library of compounds were added at 10 μM dissolved in DMSO. Reproducible characteristics of *M. marinum* growth allowed decreasing of the measurement duration from 72 down to 48 hours. The fluorescence intensities were measured for 48/72+ hours every 3 hours.

**Figure S4. Determination of the correlation between CFU counting, OD, bioluminescence and fluorescence measurements in *M. marinum* cultures.** The relationships between CFU/RFU (A), CFU/RLU (B), RFU/OD (C), RLU/OD (D), and CFU/OD (E) were characterized and coefficients of linear correlation were calculated. *M. marinum* strains either without reporter or expressing Lux or GPF were cultured as described before. The overnight cultures were diluted 10-fold into the same, fresh medium in order to obtain an OD600 of 0.2. Samples were taken at regular intervals during growth as indicated in the graphs. After incubation at 32°C, colonies on the plates were counted to determine the number of CFU.

**Figure S5. Fluorescence cumulative curves of compounds of TB-set in *A. castellanii* and *M. marinum* infection screen**. Plates GSK1 (A) and GSK2 and GSK3 (B). 5x10^4^ infected *A. castellanii* cells were transferred to the wells, in PYG medium. Compounds derived from GSK TB-set were added at 10 μM dissolved in DMSO. The fluorescence intensities were measured for 72 hours every 3 hours.

**Figure S6**. **Chemical structures of hits in cytotoxic and anti-infective assays.** The set included compounds with strong anti-infective activity in BV2 / *M. marinum* assay (A), strong anti-infective activity in *A. castellanii* / *M. marinum* assay (B).

**Figure S7**. **Amikacin effect on infection.** Comparison of BV2 infection screening with ant without 10μM in DMEM medium enriched with fetal bovine serum. BV2 cells are infected with *M. marinum* expressing GFP. 5x10^4^ infected BV2 cells were transferred to the wells, in DMEM medium supplemented with serum. Compounds derived from GSK TB-set were added at 10 μM dissolved in DMSO, +/- 10 μM amikacin. Measurement is taken 50 hours post infection.

**Figure S8.** **MIC determination in BV2/*M. marinum*-GFP assay, measured by high-content fluorescent microscopy**. BV2 cells are infected with *M. marinum* expressing GFP. Compounds derived from GSK TB-set were added at 50 μM, 25 μM, 10 μM, 2 μM, 0.4, 0.04 μM concentrations dissolved in DMSO. Displayed images of the wells correspond were taken 72 hours post infection.

**Figure S9.** **IC_50_ calculation of GSK TB-set compounds with anti-infective activity in the *A. castellanii* infection model**. 5x10^4^ infected *A. castellanii* cells were transferred to the wells, in PYG medium. Compounds derived from GSK TB-set were added at 20 μM, 10 μM, 1 μM, 0.1 µM, 0.01 μM concentrations dissolved in DMSO. Fluorescence intensities were measured for 60 post infection.

**Figure S10**. **A. Structure-activity relationship studies of Imidazo[1,2-a]pyridine-3-carboxamide compounds presented in GSK TB-set**. (A). IC_50_ calculation of GSK TB-set compounds with anti-infective activity and *A. castellanii* model. 5x10^4^ infected *A. castellanii* cells were transferred to the wells, in PYG medium. Compounds derived from GSK TB-set were added at 20 μM, 10 μM, 1 μM, 0.1 μM, 0.01 μM concentrations dissolved in DMSO. Fluorescence intensities were measured for 72 hours every 3 hours. (B). Linear regression graph based on IC_50_ values of imidazo[1,2-a]pyridine-3-carboxamide compounds that display strong antimycobacterial activity in BV2/M. marinum-GFP assay.

**Figure S11. Mechanism of action determination by generation of *M. bovis* BCG resistance mutants.** In order to identify compound modes of action, *M. bovis* BCG resistant mutants were generated at 5 times the minimal inhibitory concentration (MIC) of each compound. Four resistant isolates were identified for GSK1742694A (A and B), and ten resistant isolates were identified for GSK2043267A (C and D). Phenotypic resistance was confirmed by plating resistant isolates at 0x (B and D) and 5x MIC (A and C) along side wild type *M. bovis* BCG.

**Video 1. High-content microscopy of BV2/M. marinum-GFP infection in the presence of vehicle control**. BV2 cells are infected with *M. marinum* expressing GFP. 5x10^4^ infected BV2 cells were transferred to the wells, in DMEM medium supplemented with serum.

**Video 2**. **High-content microscopy of BV2/M. marinum-GFP infection in the presence of compound with high anti-infective activity**. BV2 cells are infected with *M. marinum* expressing GFP. 5x10^4^ infected BV2 cells were transferred to the wells, in DMEM medium supplemented with serum.

**Video 3**. **High-content microscopy of BV2/M. marinum-GFP infection in the presence of compound with high pro-infective activity**.

**Figure S1. Fluorescence cumulative curves of compounds of TB-set in *M. marinum* screen in 7H9 broth**. Plates GSK1 (A) and GSK2 and GSK3 (B). 10^5^ *M. marinum* expressing GFP were transferred to the wells. Compounds derived from GSK TB-set were added at 10 μM dissolved in DMSO. Fluorescence intensities were measured for 48/72 hours every 3 hours. Reproducible characteristics of *M. marinum* growth allowed decreasing of the measurement duration from 72 down to 35-42 hours.

**Figure S2. BV2 growth inhibition assay.** 5x10^4^ infected BV2 cells were transferred to the wells, in DMEM medium supplemented with fetal bovine serum. Either vehicle control (green frame) or the compounds derived from GSK TB-set were added at 10 μM concentrations. Measurement is taken 50 hours post infection.

**Figure S3. Fluorescence cumulative curves of compounds of TB-set in *D. discoideum*-GFPABD screen in HL5c medium.** Plates GSK1 (A) and GSK2 and GSK3 (B). 10^5^ *D. discoideum* cells expressing GFP-ABD were transferred to the wells. GSK TB-set library of compounds were added at 10 μM dissolved in DMSO. Reproducible characteristics of *M. marinum* growth allowed decreasing of the measurement duration from 72 down to 48 hours. The fluorescence intensities were measured for 48/72+ hours every 3 hours.

**
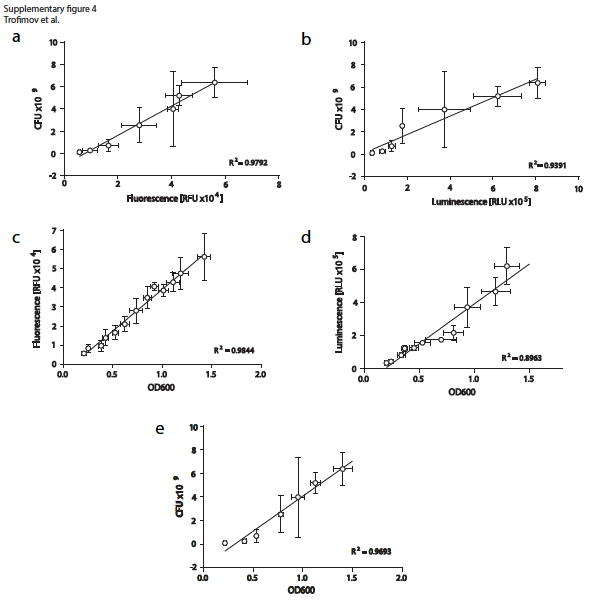
**

**Figure S4. Determination of the correlation between CFU counting, OD, bioluminescence and fluorescence measurements in *M. marinum* cultures.** The relationships between CFU/RFU (A), CFU/RLU (B), RFU/OD (C), RLU/OD (D), and CFU/OD (E) were characterized and coefficients of linear correlation were calculated. *M. marinum* strains either without reporter or expressing Lux or GPF were cultured as described before. The overnight cultures were diluted 10-fold into the same, fresh medium in order to obtain an OD600 of 0.2. Samples were taken at regular intervals during growth as indicated in the graphs. After incubation at 32°C, colonies on the plates were counted to determine the number of CFU.

**Figure S5. Fluorescence cumulative curves of compounds of TB-set in *A. castellanii* and *M. marinum* infection screen**. Plates GSK1 (A) and GSK2 and GSK3 (B). 5x10^4^ infected *A. castellanii* cells were transferred to the wells, in PYG medium. Compounds derived from GSK TB-set were added at 10 μM dissolved in DMSO. The fluorescence intensities were measured for 72 hours every 3 hours.

**Figure S6**. **Chemical structures of hits in cytotoxic and anti-infective assays.** The set included compounds with strong anti-infective activity in BV2 / *M. marinum* assay (A), strong anti-infective activity in *A. castellanii* / *M. marinum* assay (B).

**Figure S7**. **Amikacin effect on infection.** Comparison of BV2 infection screening with ant without 10μM in DMEM medium enriched with fetal bovine serum. BV2 cells are infected with *M. marinum* expressing GFP. 5x10^4^ infected BV2 cells were transferred to the wells, in DMEM medium supplemented with serum. Compounds derived from GSK TB-set were added at 10 μM dissolved in DMSO, +/- 10 μM amikacin. Measurement is taken 50 hours post infection.

**Figure S8.** **MIC determination in BV2/*M. marinum*-GFP assay, measured by high-content fluorescent microscopy**. BV2 cells are infected with *M. marinum* expressing GFP. Compounds derived from GSK TB-set were added at 50 μM, 25 μM, 10 μM, 2 μM, 0.4, 0.04 μM concentrations dissolved in DMSO. Displayed images of the wells correspond were taken 72 hours post infection.

**Figure S9.** **IC_50_ calculation of GSK TB-set compounds with anti-infective activity in the *A. castellanii* infection model**. 5x10^4^ infected *A. castellanii* cells were transferred to the wells, in PYG medium. Compounds derived from GSK TB-set were added at 20 μM, 10 μM, 1 μM, 0.1 µM, 0.01 μM concentrations dissolved in DMSO. Fluorescence intensities were measured for 60 post infection.

**Figure S10**. **A. Structure-activity relationship studies of Imidazo[1,2-a]pyridine-3-carboxamide compounds presented in GSK TB-set**. A. IC_50_ calculation of GSK TB-set compounds with anti-infective activity and *A. castellanii* model. 5x10^4^ infected *A. castellanii* cells were transferred to the wells, in PYG medium. Compounds derived from GSK TB-set were added at 20 μM, 10 μM, 1 μM, 0.1 μM, 0.01 μM concentrations dissolved in DMSO. Fluorescence intensities were measured for 72 hours every 3 hours. B. Linear regression graph based on IC_50_ values of imidazo[1,2-a]pyridine-3-carboxamide compounds that display strong antimycobacterial activity in BV2/M. marinum-GFP assay.

**Figure S11. Mechanism of action determination by generation of *M. bovis* BCG resistance mutants.** In order to identify compound modes of action, *M. bovis* BCG resistant mutants were generated at 5 times the minimal inhibitory concentration (MIC) of each compound. Four resistant isolates were identified for GSK1742694A (A and B), and ten resistant isolates were identified for GSK2043267A (C and D). Phenotypic resistance was confirmed by plating resistant isolates at 0x (B and D) and 5x MIC (A and C) along side wild type *M. bovis* BCG.
